# Assessment of fall armyworm tolerant maize hybrids for sustainable maize production in sub-Saharan Africa

**DOI:** 10.1007/s12600-025-01253-y

**Published:** 2025-02-17

**Authors:** Isaac Kodzo Amegbor, Gloria Boakyewaa Adu, Charles Nelimor, Boddupalli M. Prasanna, Yoseph Beyene, Walter Chivasa, James Gichuru Gethi, Abou Togola, Abdulai Jamal-deen, Desmond Sunday Adogoba, Jerry Nboyine, Francis Kusi, Priscilla Francisco Ribeiro, Agbesi Kwadzo Keteku, Emmanuel Wandaat, Kwabena Darkwa, Benedicta Atosona, Maryke Labuschagne

**Affiliations:** 1 CSIR-Savanna Agricultural Research Institute, P.O. Box 52, Tamale, Ghana; 2 Department of Plant Breeding, University of the Free State, Bloemfontein, South Africa; 3 International Maize and Wheat Improvement Center (CIMMYT), Nairobi, Kenya; 4 International Maize and Wheat Improvement Center (CIMMYT), Harare, Zimbabwe; 5 CSIR-Crops Research Institute, P.O. Box 3785, Kumasi, Ghana

**Keywords:** Fall armyworm, Integrated pest management, Maize hybrids, Tolerance, Sustainable maize production

## Abstract

Fall armyworm (*Spodoptera frugiperda* (J.E. Smith)) has become a significant pest to maize production, causing huge yield losses in sub-Saharan Africa. This study evaluated three non-Bt maize hybrids with tolerance to fall armyworm (FAW) along with a commercial hybrid check for yield and agronomic performance under natural FAW infestation and chemical control conditions in both on-station and on-farm trials. Significant differences were observed among the hybrids with the FAW tolerant hybrids showing reduced leaf and ear damage compared to the commercial hybrid. These hybrids also exhibited high grain yield performance, outyielding the commercial check by 197–252%. Mean grain yield under FAW infestation ranged from 6009.88 to 7117.30 kg ha^−1^ without chemical control, and even higher (8441.24 kg ha^−1^) under limited chemical control. Stepwise multiple regression analyses identified ear aspect, husk cover and ear damage as key traits accounting for 98% of the total variation in grain yield under FAW infestation. Participatory variety selection showed high farmer preference for these hybrids. The availability of these hybrids to farmers promises to enhance food security, reduce the environmental impact of insecticides, alleviate cost burdens on farmers, and increase household income.

**Supplementary Information:**

The online version contains supplementary material available at 10.1007/s12600-025-01253-y.

## Introduction

Maize cultivation is critical for food and income security in sub-Saharan Africa (SSA). In Ghana, maize accounts for at least 62% of the total annual grain production (Obour et al., 2022). It is the primary staple food crop, supplying the most calories for the Ghanaian populace. However, the emergence of fall armyworm (FAW) (*Spodoptera frugiperda* (J.E. Smith)) has posed a significant threat to maize production and productivity, threatening the country's food security. This challenge, compounded by factors such as drought, low soil nitrogen, and *Striga* infestations, has placed immense pressure on Ghana's ability to ensure a stable food supply (Gbashi et al., 2021). By January 2018, this pest had, within a span of two years, since it was first reported in Africa, spread to more than 40 African countries (Prasanna et al., 2018). The rapid expansion and establishment of FAW populations across Africa underline two critical realities; FAW demonstrates an alarming capacity to spread over vast geographical regions in short period and FAW populations can thrive all year-round under favourable tropical climates (Prasanna et al., 2021). It poses a serious threat to food security, nutritional well-being, and the livelihoods of farming households that depend on maize as staple food in Africa.

Currently, the primary recourse for FAW control in Ghana revolves around the application of pesticides. This approach, while effective, comes with a substantial financial burden, as multiple rounds of spraying, upwards of five times per crop cycle, are necessary to combat FAW infestations (Adu et al., 2024; Prasanna et al., 2021). Beyond the financial strain, the use of pesticides raises profound concerns for human health, detrimental effects on biodiversity, and the broader environment, including the potential contamination of water bodies. A study conducted by Tambo et al. (2020), which examined farmers' responses to FAW in four African countries (Ghana, Rwanda, Uganda, Zambia and Zimbabwe) revealed a significant cost of pesticide-based crop protection. In 2017, the governments of Ghana, Uganda and Zambia spent cumulatively US$14 million on distribution and education on the use of insecticides to control FAW (Day et al., 2017; Tambo et al., 2020). According to Prasanna et al. (2022), the cost of insecticide per hectare per crop season in 2018 was US$81, which increased to US$276 in 2020, primarily due to the impact of FAW. The authors further highlighted that, at the prevailing levels of FAW infestation, most farmers resorted to a minimum of six pesticide applications per crop season in 2020 (Prasanna et al., 2022). Consequently, there is a pressing need to deploy sustainable, cost-efficient, and eco-friendly strategies for the management of FAW.

Currently, all maize varieties accessible to Ghanaian farmers are susceptible to FAW. This poses a threat to food security and the livelihoods of many rural and urban households. Native genetic resistance to FAW in maize is partial, though quite significant in terms of yield protection under FAW infestation, compared to the susceptible commercial varieties. Sustainable control of FAW is best achieved when farmers combine host plant resistance with other components of integrated pest management, including good agronomic management practices, biological control, and environmentally safer pesticides (Prasanna et al., 2018, 2021, 2022).

Over the years, consumer preferences for maize have evolved, emphasizing both quantity and quality of grain. Understanding these preferences is vital for breeders, policymakers, and food industry stakeholders in meeting consumer demands and enhancing the marketability of maize-based products (Jeong & Lee, 2021; Noort et al., 2022). This interdependence between producers and consumers underscores the significance of understanding and aligning with consumer preferences in maize production. To expedite the availability of FAW-tolerant hybrids (FAWTH) to resource-limited farmers in Ghana, Savanna Agricultural Research Institute of the Council of Scientific and Industrial Research (CSIR-SARI) obtained three hybrids from CIMMYT, along with their parental lines. These materials were subjected to rigorous on-farm and on-station testing for possible release for cultivation in Ghana. This proactive measure represents a significant stride towards mitigating the FAW challenge and safeguarding the food security and livelihoods of millions of households across the country. The objectives of this study were to: (i) assess the yield performance of the three FAW-tolerant maize hybrids in comparison with a local hybrid check, (ii) involve farmers in participatory FAW-tolerant hybrids selection under their field conditions.

## Materials and methods

### Plant materials

Three FAW-tolerant hybrids viz*.* FAWTH1, FAWTH2 and FAWTH3 from CIMMYT-Kenya and a commercial local hybrid (Opeaburo) which was used as a susceptible check, were evaluated in this study. The CIMMYT maize breeding programme in Kenya has made significant progress in developing maize germplasm with tolerance/resistance to FAW since its occurrence in 2016. Over 6,000 maize genotypes were screened, leading to the identification of promising FAW-tolerant/resistant inbred lines from the Multiple insect-resistant tropical (MIRT) germplasm backgrounds. These lines were used to develop hybrids, including single-cross and three-way-cross hybrids, which were evaluated for FAW tolerance, yield, and other desirable traits. The programme later developed about 2,700 doubled haploid lines and tested over 500 hybrids, resulting in the identification of three elite FAW-tolerant tropical maize hybrids (FAWTH1, FAWTH2 and FAWTH3) with minimal leaf and ear damage and competitive grain yields. In the current study, three experiments, namely on-station evaluation, on-farm evaluation, and farmer participatory variety selection were carried out.

### Experiment one

For the on-station evaluations, field experiments were conducted at 8 locations. These were: Nyankpala (Lat. 9.390º, Long. −1.009º), Wa (Lat. 10.07751°, Long. −0.250567°, Tumu (Lat. 10.525º, Long. −1.5452º, and Damongo (Lati. 9.043º, Long. −1.811º) in the Guinea Savannah agro-ecology; Manga (Lat. 10.8622°, Long. −0.2621°) in the Sudan Savannah; and Fumesua (Lat. 5.05491º, Long. −2.48821º), Ho (Lat. 6.7430º, Long. 0.4654º) and Dzakiti, in the Forest-Savannah Transition zone. Characteristics of the soil and rainfall for some of these locations are shown in Tables 1 and 2, respectively. The three hybrids together with the local hybrid check were arranged in a randomized complete block design with three replications under natural FAW infestation (without the use of insecticides). The same set of hybrids was evaluated under chemical control, hereafter referred to as “control plots” in this experiment on the same field. To avoid chemical drift to the trial under natural FAW infestation, 5 m was left between the two plots at all locations. For the chemical control plots, limited spraying was conducted in all fields using EmaStar 112 EC (250 ml)® (Emamectin benzoate and acetamiprid) insecticide at a rate of 25 ml per 16 L knapsack. Since the aim was to reduce the use of chemical insecticides, the FAW-tolerant hybrid plots were sprayed twice in the control plots at 14 and 28 days after planting, while susceptible hybrid plots were sprayed 6–7 times, depending on the location and FAW action threshold. FAW action threshold is the point at which the FAW population becomes large enough to require control measures to prevent significant yield losses. Below this threshold, the damage caused by FAW may be minimal, and control measures may not be necessary (Prasanna et al., 2018). The hybrids, experimental design, and replications were the same at all locations. Each hybrid was planted in a four-row plot of 5 m length with intra row spacing of 0.25 m and inter-row spacing of 0.75 m.

**Table 1:** Physico-chemical properties of the soils at some of the experimental sites

Location	Textural class	Total nitrogen (%)	Organic carbon (%)	Organic matter (%)	Ca (Cmol/Kg)	Mg (Cmol/Kg)		K (Cmol/Kg)
Nyankpala	Sandy loam	0.06	0.59	3.12	10.6	1.90		0.14
Damongo	Loamy fine sand	0.07	0.83	4.26	30.8	4.60		0.18
Yendi	Fine sand	0.11	0.62	4.60	13.2	4.80		0.16
Manga	Sandy	0.07	0.12	3.62	12.2	2.40		0.12
Fumesua	Fine sand	0.07	1.230	3.84	8.40	0.60		0.22
Ho	Sandy loam	0.10	1.11	3.00	9.23	3.72		0.23
Location	Na (Cmol/Kg)	Al (Cmol/Kg)	H (Cmol/Kg)	P (mg/Kg)	Sand (%)	Clay (%)	Silt (%)	pH
Nyankpala	0.23	0.41	0.21	5.705	69.4	9.40	20.200	5.28
Damongo	0.80	0.67	0.33	32.28	84.0	10.00	6.00	5.78
Yendi	0.67	0.67	0.50	9.33	88.24	4.00	7.76	5.32
Manga	0.41	0.34	0.32	7.987	85.64	5.36	9.00	5.24
Fumesua	0.18	0.50	0.30	18.16	90.0	6.12	3.88	6.56
Ho				10.30				6.40

**Table 2:** Mean monthly rainfall amount (mm) at some of the experimental sites during the period of the study

Year	Manga		Nyankpala		Fumesua		Damongo		Yendi	
	Minimum	Maximum	Minimum	Maximum	Minimum	Maximum	Minimum	Maximum	Minimum	Maximum
2020/2021	145.95	275.03	122.50	157.89	110.10	223.40	112.33	160.66	99.88	140.33
2021	126.91	352.83	149.98	288.41	109.99	205.58	153.20	292.32	120.22	210.99
2022	168.69	277.79	93.45	165.11	114.20	394.80	97.54	187.33	98.54	170.23

### Experiment two

The on-farm experiment consisted of farmer and researcher managed trials. For the farmer managed trials (baby trial), each farmer served as a replicate while the researcher managed trials (mother trial) used a randomized complete block design with three replications as described under experiment one. The mother trial was conducted in 10 locations while the baby trial was conducted by a total of 120 farmers, 12 farmers in each location. The plot size was 10 m by 10 m under farmer and researcher managed conditions per variety. The agronomic practices applied in these on-farm trials were similar to those in the on-station trials where there was no use of insecticides to ensure consistency and comparability in the evaluation process.

For experiments one and two, two seeds were planted per hill and thinned to one plant per hill at two weeks after planting to obtain estimated plant population of 53,333 plants ha^−1^. Weeds were controlled both chemically (using pre- and post-emergence herbicides) and manually using a hoe. Basal fertilizer was applied at a rate of 60 kg N ha^−1^ and 60 kg P_2_O_5_ ha^−1^ as basal fertilizer at two weeks after planting (WAP) and top-dressed with additional N (urea) at 60 kg N ha^−1^ at four WAP.

### Experiment three

Participatory variety selection (PVS) trials were conducted for the three FAW-tolerant hybrids alongside the local check. The PVS trials were conducted in the Guinea and Sudan Savannah, as well as the Forest and Forest-Savannah Transition agroecological zones in 2021 and 2022. A total of 245 farmers participated in the varietal selection with a gender-balanced composition of 40.41% female to 59.59% male participants. The participants were allowed to go through the field. Each participant selected his/her preferred variety and assigned reasons for the selection.

### Traits measured

In the on-station and on-farm trials, observations were made on plot basis for days to 50% silking (DS) and anthesis (DA), plant height (PH, cm), ear height (EH, cm). Plant aspect (PA) was rated on a scale 1 to 9, where a score of 1 represents an excellent plant type characterized by optimal growth habits, vigorous development, and desirable morphological features, while a score of 9 indicates a poor plant type exhibiting suboptimal growth, weak development, and undesirable morphological characteristics. Ear aspect (EA) was rated on a scale 1 to 9, where a score of 1 denotes ideal ear characteristics, including cleanliness, uniformity, large size, and well-filled kernels, while a score of 9 signifies ears with undesirable features, such as rotten or disease kernels, variability in ear size, shape, and uneven kernel filling Amegbor et al. (2017). FAW infestations were scored on plot basis for leaf damage at 70 days after planting (DAP) using a scale of 1 to 9, where 1 = minimal or no damage of leaves and 9 = virtually all leaves damaged (Davis et al., 1992). Additionally, ear damage was scored using a scale of 1 to 9, where 1 = undamaged ears and 9 = virtually all grains damaged on the ear. Grain yield was computed from shelled grain weight per plot, adjusted to a grain moisture of 12% and converted to kg ha^−1^. At harvest, farmers were invited to provide their assessments on ear size, grain filling, ear damage, number of ears per plant, and grain yield.

### Data analysis

Data from the on-station and on-farm experiments were subjected to analysis of variance (ANOVA) using PROC GLM in SAS (SAS Institute, 2011 version 9.4) with testing for heterogeneity of variances among the genotypes. Best linear unbiased predictors (BLUPs) were estimated for all traits along with least significance differences (LSD) at each location and combined locations to test the significance of variance components and to determine genotype by environment interaction effects. Further phenotypic correlations were estimated to determine trait associations. The ANOVA model used was as described of Alwala et al. (2010)

1 $${Y}_{ij}= \mu +G\_i+ {E}_{j}+ {GE}_{ij}$$

In the equation, ${Y}_{ij}$ represents the response variable of the *i*_*th*_ genotype at the *j*_*th*_ location, *μ* denotes the overall mean, *G* signifies the main effect of genotype *i, E* stands for the main effect of environment *j*, and *GE* represents the error term, which in this case, is confounded with the genotype-by-environment interaction effect.

Correlation and path analysis were performed following the descriptions by Ali et al. (2013) and Arminian et al. (2008), respectively.

The selection index (SI) described below was used to select hybrids with high grain yield potential and superior agronomic performance across locations and years with integration of several traits:

2 $$SI=\left[\left(2 x GY\right)+EPP-ASI-PA-EA-FAWLD-FAWED\right]$$

where GY represents the average grain yield of a hybrid across various environments, PA denotes the average plant aspect of a hybrid across those environments, EA signifies the average ear aspect of a hybrid across the same environments, EPP represents the average number of ears per plant of a hybrid across these environments, and ASI is the average anthesis-silking interval of a hybrid across these environments.

For the PVS, data collected were analysed using Statistical Package for the Social Sciences (SPSS) edition 20 (IBMCorp., 2020).

## Results

### On-station multi-locational experiment

The three FAW hybrids (FAWTH1, FAWTH2 and FAWTH3) significantly outperformed the local hybrid check at all the locations, except Tumu (Supplementary Table 1). Mean grain yield of all the 4 hybrids at the respective sites ranged from 3173.96 kg ha^−1^ at Manga to 6388.72 kg ha^−1^ at Tumu. Except for the trial at Tumu, each FAWTH had grain yield higher than the overall mean at all the locations. At these locations, the test hybrids yielded at least 2630.00 kg ha^−1^ more than the local check, translating to a yield advantage of over 200%. The genotypic variance for grain yield was higher than the variance due to environment and genotype by environment interaction at all the test locations, except in Tumu. The broad sense heritability of grain yield was low at Tumu (0.41) but very high at the other locations (above 0.90). In the combined analyses across locations, the FAWTH differed significantly from the local check for grain yield and other measured traits (Supplementary Table 2). Compared to the local check, all three FAWTH recorded desirable plant and ear aspect scores along with reduced leaf and ear damage (Supplementary Table 2). Hybrid FAWTH3 recorded the highest grain yield of 7117.30 kg ha^−1^. The grain yield of FAWTH1 and FAWTH2 were comparable. The yield advantages of the FAWTH over the local check were approximately 197%, 201% and 252% for FAWTH1, FAWTH2 and FAWTH3, respectively.

Also, in the combined ANOVA, the mean yield of sprayed trials relative to the non-sprayed trials showed that overall yield reduction due to FAW infestation was approximately 38% (Table 3 and Supplementary Table 3). Grain yield reduction was low for the FAWTH hybrids, ranging from 18.6% for FAWTH3 to 24.19% for FAWTH2. In contrast, FAW reduced the grain yield of the local check by over 200%. The low yield reduction of the FAWTH was accompanied by desirable plant and ear aspect scores along with reduced leaf and ear damage in both insecticide sprayed and natural FAW infested trials, resulting in high positive index selection values. The reverse was observed for the local check which had a high negative index selection value of −15.2, meaning the check variety is indeed susceptible to FAW infestation (Table 3). However, in the insecticide sprayed trials, the test hybrids and the local check were statistically not different for ASI, PA and EPP but varied significantly from each other for the same traits in the trials conducted under natural FAW infestation, which indicate the differential response of the hybrids to FAW infestation.

**Table 3:** Grain yield and selected agronomic traits of three fall armyworm tolerant maize hybrids and a local check across natural infestation (NI) and insecticide sprayed (SP) conditions for on-station trials

Entry	GY (kg ha^−1^)		ASI		PH (cm)		EH (cm)		PA (1–9)		EA (1–9)		EPP		FAWD		YR(%)	BI
	NI	SP	NI	SP	NI	SP	NI	SP	NI	SP	NI	SP	NI	SP	Leaf	Ear		
FAWTH1	6009.88	7225.29	1.4	1.1	189.25	216.78	102.00	110.22	3.25	2.67	3.92	2.61	0.93	0.96	2.58	2.21	20.22	12.3
FAWTH2	6078.84	7549.49	1.6	0.9	188.71	219.00	94.50	111.44	3.00	2.78	3.92	2.67	0.9	0.95	2.6	2.2	24.19	14.4
FAWTH3	7117.30	8441.24	1.9	1.0	180.13	212.39	96.96	106.11	3.33	2.58	3.63	2.28	0.9	0.95	2.3	2.1	18.60	26.7
Opeaburo	2022.93	6098.86	1.8	1.0	192.25	203.78	99.67	103.44	5.54	2.72	5.96	3.22	0.9	0.93	4.6	4.5	201.49	−15.2
R-Square	0.92	0.97	0.5	0.6	0.84	0.79	0.78	0.69	0.79	0.55	0.73	0.82	0.7	0.44	0.8	0.9		
CV (%)	8.87	6.22	54.3	48.9	8.98	5.45	14.89	8.25	20.00	21.59	20.27	17.68	8.7	12.91	26.0	17.2		
LSD	533.08	655.51	0.9	0.5	16.85	11.60	14.63	8.89	0.71	0.58	0.83	0.48	0.1	0.12	0.8	0.5		
Mean	5307.24	7328.72	1.7	1.0	187.58	212.99	98.28	107.81	3.53	2.69	4.10	2.69	0.9	0.95	3.0	2.8		
Location	***	***	***	ns	***	***	***	*	***	ns	***	***	***	ns	***	***		
Entry	***	***	ns	ns	ns	**	ns	*	***	ns	***	***	*	ns	***	***		
G x E	***	**	ns	ns	*	**	ns	*	***	ns	***	***	**	ns	*	***		
Heritability	0.91	0.97	0.78			0.75			0.59		0.06	0.76	0.28		0.94	0.92		

^**†**^ GY = Grain yield (kg ha^−1^); ASI-anthesis silking interval; *PH* = plant height; EH-ear height; *PA* = plant aspect; *EA*-ear aspect; *EPP*-ears per plant; *FAWD* = FAW damage on leaf and ear; *P ≤ 0.05, **P ≤ 0.01, *P* ≤ 0.001, ns – not significant; *YR* = Yield reduction under fall armyworm infestation or uncontrolled environment; *G x E* = Genotype by environment interaction; *LSD* = Least significant difference, *CV* = coefficient of variation; *BI* = Base index

### Correlation and trait association under FAW infestation

**Fig. 1:**
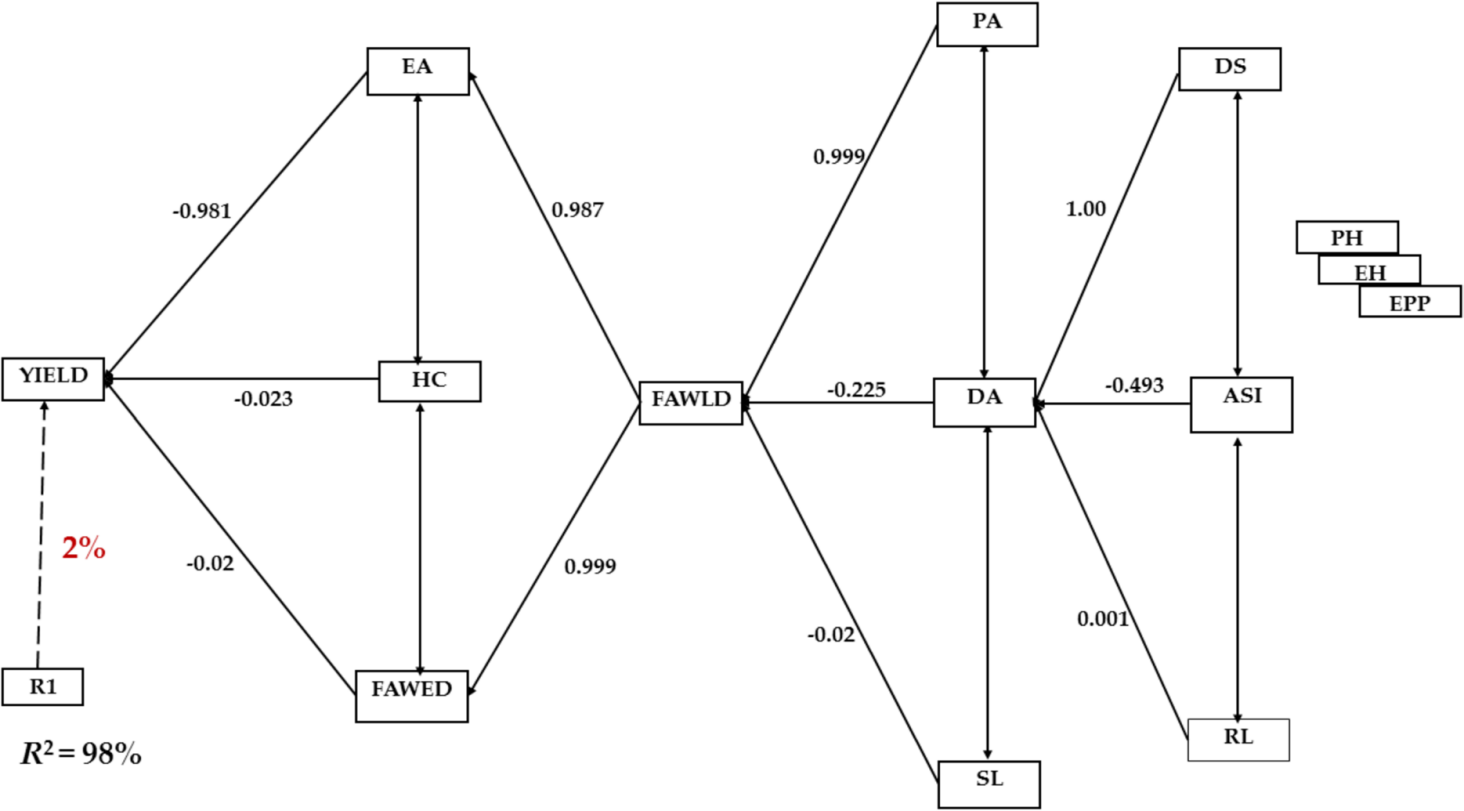
Path analysis diagram showing the relationship between grain yield and measured traits of the three FAW-tolerant maize hybrids and local check under fall armyworm infestation. **†** Yield = Grain yield (kg ha^−1^); DA = days to 50% anthesis; DS = days to 50% silking; ASI = anthesis silking interval; PH = plant height; EH = ear height; PA = plant aspect; EA = ear aspect; EPP = ears per plant; HC = husk cover; RL = root lodging; SL = stalk lodging; FAWLD = leaf damage by FAW; FAWED = ear damage by FAW

Correlation analysis showed that days to anthesis and silking, stem lodging, and plant and ear aspects, as well as leaf and ear damage had low to moderate negative correlation with grain yield (Table 4). The highest positive correlation was observed between leaf and ear damage (*r* = 0.73, *p* < 0.01). Stepwise multiple regression analysis categorized the traits under FAW infestation into four main orders according to their relationship with grain yield (Fig. 1). The first order traits which accounted for 98% of the variation in grain yield included ear aspect, husk cover and ear damage. Amongst these traits, ear aspect had the strongest relationship (−0.981) with grain yield**.** Only leaf damage was classified into the second order and had indirect effect on grain yield through ear aspect and ear damage. The three traits classified into the third order (plant aspect, days to 50% anthesis and stem lodging) each contributed to grain yield through leaf damage. The fourth order traits with indirect effect on grain yield were days to 50% silking, anthesis-silking interval, and root lodging. The remaining traits (plant and ear heights, and ears per plant) had no direct or indirect effect on grain yield.

**Table 4:** Phenotypic correlation among traits of fall armyworm tolerant hybrids and a local check under natural fall armyworm infestation for on-station trials

	DA	DS	ASI	PH	EH	RL	SL	HC	PA	EA	GY	EPP	FAWLD	FAWED
DA	1													
DS	0.96**	1												
ASI	0.32**	0.57**	1											
PH	0.01	0.01	−0.02	1										
EH	0.02	0.02	0.02	0.82**	1									
RL	0.03	−0.01	−0.13	−0.1	−0.01	1								
SL	0.26*	0.23*	0.02	0.17	0.11	−0.17	1							
HC	−0.02	−0.02	−0.01	−0.23*	−0.28**	−0.1	0.06	1						
PA	−0.06	−0.03	0.07	−0.31**	−0.25*	0.09	−0.19	−0.15	1					
EA	0.07	0.07	0.02	−0.29**	−0.30**	0.14	−0.1	0.07	0.31**	1				
GY	−0.41*	−0.37**	−0.06	0.18	0.31**	0.16	−0.32**	−0.12	−0.26*	−0.27**	1			
EPP	0.01	−0.04	−0.17	−0.28*	−0.29**	0.19	0.016	0.11	0.02	0.11	0.03	1		
FAWLD	−0.01	−0.03	−0.05	−0.22*	−0.22*	−0.08	0.05	−0.07	0.56**	0.39**	−0.51**	−0.11	1	
FAWED	−0.05	−0.04	0.02	−0.03	−0.07	−0.11	0.09	−0.13	0.44**	0.24*	−0.50**	−0.23*	0.73**	1

^**†**^ *GY* = Grain yield; *DA* = days to 50% anthesis; *DS* = days to 50% silking; *ASI* = anthesis silking interval; *PH* = plant height; *EH* = ear height; *PA* = plant aspect; *RL* = root lodging; *SL* = stalk lodging; *EA* = ear aspect; *EPP* = ears per plant; *HC* = husk cover; *FAWLD* = leaf damage by fall FAW; *FAWED* = ear damage by fall FAW; *P ≤ 0.05, **P ≤ 0.01, *P* ≤ 0.001, ns – not significant

### Average grain yield and agronomic performance of the fall armyworm tolerant maize varieties across locations for mother and baby trials

The grain yield of the three FAWTH and local check showed significant difference at the individual locations (Supplementary Table 4). The highest average grain yield (6743.85 kg ha^−1^) was observed at Tumu while Manga had the lowest mean grain yield of 3315.60 kg ha^−1^. At each location, the three FAW-tolerant hybrids out-performed the local check by at least 20%. Among them, hybrid FAWTH3 consistently achieved the highest yield while the yield of hybrids FAWTH1 and FAWTH2 were similar at all the locations. In both the mother and baby trials, the FAWTH and local check varied significantly for grain yield and majority of the measured traits (Table 5).

**Table 5:** On-farm grain yield and agronomic traits of three fall armyworm tolerant varieties and a local check across 10 locations in Ghana

Entry	Grain yield				Agronomic traits												
	Mother trial	Baby trial	Mean	YAD (%)	DA	DS	ASI	PH	EH	RL	SL	HC	PA	EA	EPP	FAWLD	FAWED
FAWTH1	6428.43	5954.69	6191.56	137.33	56.0	57.3	1.3	185.99	86.17	1.00	0.52	1.70	2.81	2.00	1.01	2.22	1.96
FAWTH2	5913.21	4075.69	4994.45	91.45	55.7	56.7	1.1	183.75	88.69	1.30	0.78	1.85	3.22	2.19	1.01	2.44	2.19
FAWTH3	7578.96	6931.01	7254.99	178.10	55.8	57.1	1.3	178.80	83.95	1.70	1.07	1.81	2.63	1.56	1.02	2.07	1.93
Opeaburo	3184.71	2032.89	2608.80	0.00	56.5	57.6	1.1	174.36	83.68	1.48	0.89	1.70	4.56	4.93	0.99	4.59	4.41
R-Square	0.98	0.94	0.96		0.9	0.9	0.8	0.95	0.90	0.79	0.75	0.73	0.88	0.93	0.89	0.90	0.85
CV	7.19	8.32	7.75		2.6	2.7	44.3	5.20	8.48	70.71	61.40	22.49	17.27	18.66	5.42	20.33	26.94
LSD	415.05	321.63	368.34		1.4	1.5	0.5	9.40	7.26	0.97	0.50	0.40	0.57	0.50	0.05	0.58	0.71
Mean	5776.33	4748.57	5262.45		56.0	57.2	1.2	180.73	85.62	1.37	0.81	1.77	3.31	2.67	1.01	2.83	2.62
Location	***	***			**	**	***	***	***	***	***	***	***	*	****	***	***
Genotype	***	***			ns	ns	ns	***	*	***	**	ns	***	***	****	***	***
G x E	**	**			ns	ns	ns	***	***	***	**	***	**	***	****	**	ns
Heritability	0.99	0.89	0.94		0.84	0.77	0.26			0.53			0.90			0.98	0.98

^**†**^ *GY* = Grain yield (kg ha^−1^); *DA* = days to 50% pollen shed; *DS* = Days to silking; ASI-anthesis silking interval; *PH* = plant height; EH-ear height; *PA* = plant aspect; *EA*-ear aspect; *EPP*-ears per plant; *HC*-husk cover; *Rot* = ear rot; *EA*-ear aspect; *RL* = root lodging, *SL* = Stalk lodging; *FAWLD* = leaf damage by FAW; *FAWED* = ear damage by FAW; *P ≤ 0.05, **P ≤ 0.01, *P* ≤ 0.001, ns – not significant; *YAD* = yield advantage over the commercial check; *G x E* = Genotype by environment interaction; *LSD* = Least significant difference, *CV* = coefficient of variation

The mother trial, with an average grain yield of 5776.33 kg ha^−1^ out-yielded the baby trial, which had an average yield of 4748.57 kg ha^−1^ by 21.64%. Among the test hybrids, FAWTH3 produced the highest grain yield under both trials, followed by FAWTH1 and FAWTH2 with mean yields of 7254.99, 6191.56, and 4994.45 kg ha^−1^, respectively. The mean grain yield of the local check was less than 3000 kg ha^−1^ across locations, which was at least 92% lower than the FAW-tolerant hybrids. The high yield advantage of the FAWTH over the local check was largely attributed to the excellent plant and ear aspect scores along with minimal leaf and ear damage. Grain yield had moderate to strong negative association with plant aspect (*r* = −0.45, *p* < 0.001), ear aspect (*r* = −0.71, *p* < 0.001), leaf damage (*r* = −0.56, *p* < 0.001) and ear damage (*r* = −0.61, *p* < 0.001). Plant and ear aspect scores were positively correlated with both leaf and ear damage. The association between leaf damage and ear damage was strong and positive (*r* = 0.79, *p* < 0.001).

### Participatory varietal selection

**Fig. 2:**
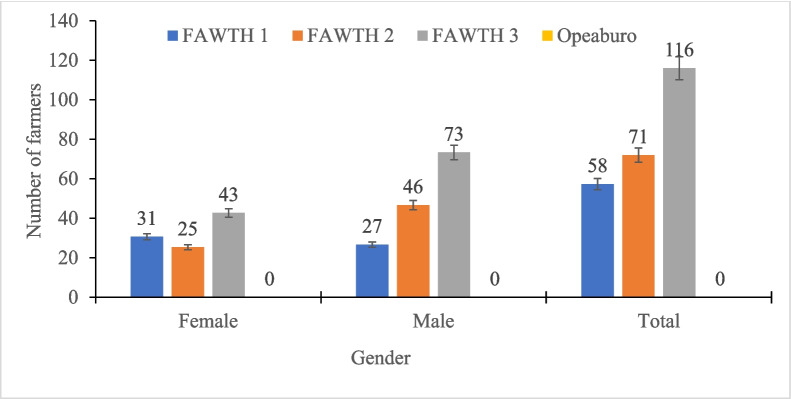
Farmers preferences for maize hybrids evaluated under natural fall armyworm infestation

**Fig. 3:**
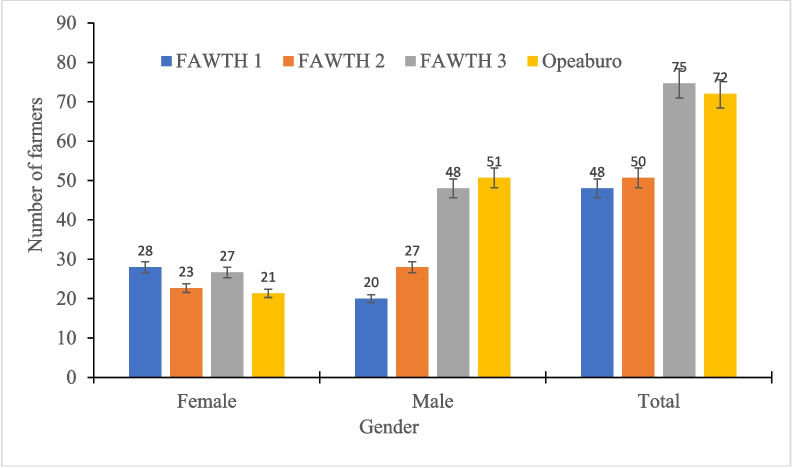
Farmers preferences for maize hybrid in trials where fall armyworm was controlled by means of insecticide applications

**Fig. 4:**
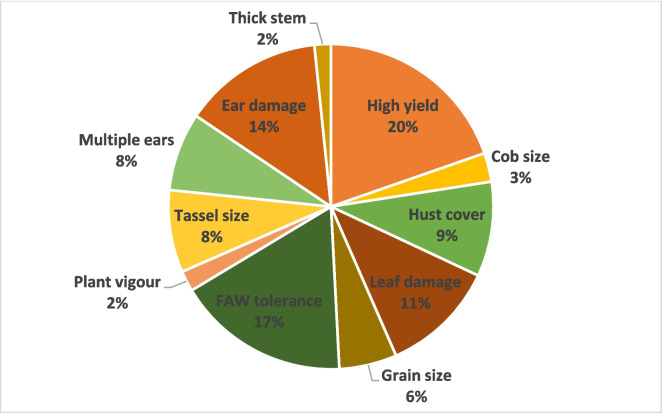
Top traits preferred by the participating farmers during variety selection

Figures 2 and 3 show the farmers’ varietal selections made during the field day. The results showed that the number of farmers who preferred FAWTH under natural infestation where there was no chemical intervention ranged from 25 to 43 for the women and 27 to 73 for the men (Fig. 2). Overall, FAWTH3 was the most preferred hybrid by 116 farmers, followed by FAWTH2 preferred by 71 farmers and FAWTH1 by 58 farmers. None of the farmers preferred the local check under FAW infestation (Fig. 2). However, in the FAW control plots (insecticide sprayed), FAWTH1 and the local check were the most and least preferred hybrids by the women, respectively (Fig. 3). Interestingly, most of the males (51) preferred the local check, followed by FAWTH3 (48). Overall, FAWTH3 (75 farmers) was the most popular test hybrid preferred by the farmers when sprayed with insecticide, followed by the local check (72 farmers), FAWTH2 (50 farmers) and FAWTH1 (48 farmers) (Fig. 3). Reduced leaf, tassel and ear damage, tolerance to FAW, proliferation, high grain yield, and stem vigour, ear size, and desirable husk cover were cited by the farmers as the major reasons for their selection of the varieties under natural FAW infestation (Fig. 4).

## Discussion

The genotypic variance for grain yield exceeded the variance due to environmental factors and genotype by environment interactions at all test locations except Tumu. Broad-sense heritability of grain yield was high at most locations, indicating a strong genetic control of yield performance in these environments.

The assessment of genetic resources is a crucial foundation for the development of superior products, particularly in the context of addressing production constraints. In this investigation, maize hybrids were evaluated for tolerance to FAW to identify elite genotypes possessing desirable traits. This was to understand the genetic potential of these hybrids in mitigating the detrimental effects of FAW infestation on maize grain yield and agronomic traits. The performance of the tested maize hybrids revealed superiority of the three FAW test hybrids (FAWTH1, FAWTH2, and FAWTH3) over the local hybrid check at all the locations. The superior performance of the FAW-tolerant hybrids over the local check in terms of grain yield and most agronomic traits, implied that the hybrids examined exhibited substantial grain yield advantages over the local check, indicating their significant potential for mitigating the negative impact of FAW infestation on maize yields. The yield recorded for the FAWTH hybrids in this experiment is within the range of 2.61 – 9.69 t ha^−1^ reported by Kamweru et al. (2023) for experiments under FAW infestation.

The FAWTH exhibited desirable ratings for plant and ear aspects, along with minimal leaf and ear damages, contributing to their superior yield performance compared to the local check. The results indicate that the leaf tissue and photosynthetic area of the FAWTH exhibited reduced damage from FAW infestation compared to the susceptible check, thereby preserving photosynthetic capacity, and contributing to enhanced grain yield in the test hybrids. This suggests that the FAWTH possess increased tolerance to FAW damage, likely due to enhanced defence mechanisms or physical traits that reduce herbivory, resulting in improved agronomic performance corroborating the earlier studies (Lima et al., 2022; Overton et al., 2021).

Additionally, the study evaluated the impact of FAW infestation on grain yield in both insecticide sprayed and natural FAW infested trials. The differential response of the FAWTH and the local check to FAW infestation was evident in both sprayed and non-sprayed trials, as indicated by their significantly different performance for various traits. FAW infestation was found to cause grain yield reduction by 38%, with the local check suffering the most severe reduction of over 200%. Comparing the FAWTH under sprayed and non-sprayed conditions, these genotypes exhibited lower yield reductions ranging from 18.6% to 24.19%, indicating their resilience to FAW damage. The observed yield reduction in the present study is comparable to the yield losses recorded by Tambo et al. (2020) where FAW reduced grain yield between 21–53%. Job et al. (2022) under severe infestation by FAW, yield losses could be as high as 53%. Our study affirmed the importance of genetic tolerance mechanisms in combating FAW infestation and highlighted the potential of the FAWTH for maintaining yield stability under FAW pressure.

The association among traits under FAW infestation is crucial to identify secondary traits that can be used in indirect selection for FAW tolerance. The results of correlation analysis in this study showed the intricate relationships between various agronomic traits and grain yield under FAW infestation. The observed weak to moderate negative correlations between days to anthesis and silking, stem lodging, as well as leaf and ear damage, with grain yield suggested these traits may have negative effects on grain yield. In contrast, the high positive correlation found between leaf and ear damage implied that these two traits could be improved simultaneously for increased tolerance to FAW. The stepwise multiple regression analysis provided further insights into the hierarchy of traits based on their contributions to the total variation in grain yield under FAW infestation. The first-order traits (ear aspect, husk cover, and ear damage), collectively explained a significant portion (98%) of the variation in grain yield. This observation implied that genotypes selected under FAW infestation based on these traits would be more productive. Therefore, the inclusion of ear aspect and ear damage in the FAW tolerant base index is justified. Among the first order traits, ear aspect exhibited the strongest relationship with grain yield, indicating its importance as a primary determinant of yield under FAW pressure. Leaf damage was classified as a second-order trait, indicating its indirect effect on grain yield through its influence on ear aspect and ear damage. This underlines the impact of FAW damage on different plant components and ultimately on yield. The third-order traits, comprising plant aspect, days to 50% anthesis, and stem lodging, were found to contribute to grain yield primarily through their association with leaf damage. This observation suggested that genotypes with high folia damage by FAW have weakened stems as FAW larvae may bore holes into the stem of susceptible varieties predisposing them to stem lodging. Furthermore, the stem become weak because damaged leaves imply a reduced area for photosynthesis to occur and thus, plant vigour is reduced. Consequently, the poorly nourished plants have weak stems. The implication is that indirect selection for grain yield can be achieved by focusing on third-order traits such as plant aspect, days to 50% anthesis, and stem lodging, which are associated with leaf damage. This suggests that breeders can use these traits as indirect selection criteria to improve grain yield, rather than directly selecting for grain yield. This approach can be particularly useful in breeding programmes where direct selection for grain yield is challenging or inefficient.

The participatory varietal selection study highlighted the importance of considering farmer preferences and priorities in maize breeding programs. The inclusion of both male and female participants ensured a diverse range of perspectives and considerations in the selection process. The high preferences for FAW-tolerant hybrids under FAW infestation than the check and a fair preference for the check under sprayed condition is consistent with previous research highlighting the importance of involving farmers in varietal selection processes to ensure that newly developed varieties meet the specific needs and preferences of end-users (Adu et al., 2021). Therefore, by aligning breeding objectives with end-user preferences, breeders can develop cultivars that not only exhibit superior agronomic performance but also meet the specific needs and challenges faced by farmers in their farming practices. The preference for FAWTH signified the importance of developing cultivars with inherent resistance or tolerance to pests, such as the FAW. By selecting hybrids with desirable traits, farmers can potentially minimize yield losses and reduce reliance on chemical pesticides, thereby promoting sustainable agricultural practices.

## Conclusion

The test hybrids demonstrated noteworthy tolerance to the FAW through reduced leaf and ear damage. The high grain yield, reduced leaf and ear damage, and adaptability across diverse agro-ecologies suggested the potential of these hybrids for widespread adoption as well as eliminating the need for extensive chemical interventions. The hybrids offer a sustainable solution to the agricultural challenges posed by FAW. Furthermore, the responses from farmers through participatory variety selection highlight the feasibility and acceptability of these hybrids. These efforts are vital for ensuring a stable and secure food supply in the face of challenges posed by invasive pests.

## Supplementary Information

Below is the link to the electronic supplementary material.

Supplementary file1 (DOCX 39 KB)

## Data Availability

Data will be made available on request.
