# Supplementary material for: Assessment of fall armyworm tolerant maize hybrids for sustainable maize production in sub-Saharan Africa
Source: Phytoparasitica. 2025 Feb 17;53(2):29. doi: 10.1007/s12600-025-01253-y (PMC11832672; doi:10.1007/s12600-025-01253-y)
Supplement: Supplementary file 1 — Supplementary file1 (DOCX 39 KB) [file 12600_2025_1253_MOESM1_ESM.docx]

**Supplementary Table 1.** On-station grain yield (kg ha^-1^) of three fall armyworm tolerant maize hybrids and a local check evaluated across eight locations in Ghana

| Genotype | Damongo | Fumesua | Ho | Manga | Nyankpala | Tumu | Wa | Yendi |
| --- | --- | --- | --- | --- | --- | --- | --- | --- |
| FAWTH1 | 5799.91 | 6692.79 | 7223.94 | 3680.52 | 5157.25 | 6396.85 | 6612.84 | 6534.86 |
| FAWTH2 | 6183.04 | 6335.09 | 6777.64 | 3716.04 | 5448.16 | 6245.11 | 6125.31 | 6936.98 |
| FAWTH3 | 7799.30 | 7730.15 | 7013.92 | 4248.84 | 7611.32 | 6342.66 | 7296.65 | 7457.38 |
| Opeaburo | 2134.40 | 2190.42 | 2071.21 | 1050.45 | 1125.32 | 6570.26 | 2743.04 | 1848.90 |
| Heritability | 0.96 | 0.96 | 0.99 | 0.95 | 0.95 | 0.41 | 0.93 | 0.89 |
| Genotype Variance | 932567.98 | 1142046.79 | 980792.52 | 468114.26 | 2450997.14 | 45610.25 | 4029742.46 | 517622.66 |
| Residual Variance | 117393.48 | 139635.64 | 38008.68 | 67566.37 | 422674.62 | 198881.59 | 936361.54 | 192870.76 |
| Grand Mean | 5479.16 | 5737.11 | 5771.68 | 3173.96 | 4835.51 | 6388.72 | 5694.46 | 5694.53 |
| LSD | 670.61 | 731.81 | 387.01 | 507.26 | 1263.10 | 568.82 | 1862.49 | 827.53 |
| CV | 5.35 | 5.76 | 2.99 | 7.27 | 6.73 | 6.98 | 8.02 | 6.56 |

NB: LSD = Least significant difference, CV = coefficient of variation

**Supplementary Table 2.** On-station grain yield and agronomic traits of fall armyworm tolerant varieties and a local check combined across eight locations in Ghana under natural fall armyworm infestation

| Entry | GY | YAD | DA | DS | ASI | PH | EH | RL | SL | HC | PA | EA | EPP | FAWLD | FAWED |
| --- | --- | --- | --- | --- | --- | --- | --- | --- | --- | --- | --- | --- | --- | --- | --- |
| FAWTH1 | 6009.88 | 197.09 | 58.60 | 60.00 | 1.40 | 189.25 | 102.00 | 0.46 | 0.63 | 2.13 | 3.25 | 3.92 | 0.90 | 2.50 | 2.20 |
| FAWTH2 | 6078.84 | 200.50 | 58.00 | 59.60 | 1.60 | 188.71 | 94.50 | 0.88 | 1.04 | 2.33 | 3.00 | 3.92 | 0.90 | 2.60 | 2.20 |
| FAWTH3 | 7117.30 | 251.83 | 59.00 | 60.90 | 1.90 | 180.13 | 96.96 | 1.33 | 0.83 | 2.17 | 3.33 | 3.63 | 0.90 | 2.30 | 2.10 |
| Opeaburo | 2022.93 | 0.00 | 58.90 | 60.70 | 1.80 | 192.25 | 99.67 | 1.04 | 1.00 | 1.96 | 5.54 | 4.96 | 0.90 | 5.60 | 5.50 |
| R-Square | 0.92 |  | 0.90 | 0.90 | 0.50 | 0.84 | 0.78 | 0.60 | 0.67 | 0.52 | 0.79 | 0.73 | 0.70 | 0.80 | 0.90 |
| CV | 8.87 |  | 2.20 | 2.40 | 54.30 | 8.98 | 14.89 | 164.62 | 80.19 | 20.84 | 20.00 | 20.27 | 8.70 | 26.00 | 17.20 |
| LSD | 533.08 |  | 1.30 | 1.40 | 0.90 | 16.85 | 14.63 | 1.53 | 0.70 | 0.45 | 0.71 | 0.83 | 0.10 | 0.80 | 0.50 |
| Mean | 5307.24 |  | 58.60 | 60.30 | 1.70 | 187.58 | 98.28 | 0.93 | 0.88 | 2.15 | 3.53 | 4.10 | 0.90 | 3.00 | 2.80 |
| Location | *** |  | *** | *** | *** | *** | *** | ** | *** | *** | *** | *** | *** | *** | *** |
| Entry | *** |  | * | ** | ns | ns | ns | ns | ns | * | *** | *** | * | *** | *** |
| G x E | *** |  | *** | *** | ns | * | ns | ns | * | * | *** | *** | ** | * | *** |
| Heritability | 0.91 |  |  |  | 0.78 |  |  |  | 0.16 | 0.26 | 0.59 | 0.06 | 0.28 | 0.94 | 0.92 |

**†** GY= Grain yield (kg ha^-1^); DA = days to 50% pollen shed; DS = Days to 50 silking; ASI-anthesis silking interval; PH = plant height; EH-ear height; PA = plant aspect; EA-ear aspect; EPP-ears per plant; HC = husk cover; Rot = ear rot; EA-ear aspect; RL = root lodging, SL = Stalk lodging; FAWLD = leaf damage by FAW; FAWED = ear damage by FAW; *P≤0.05, **P≤0.01, P≤0.001, ns – not significant ; YAD = yield advantage over the commercial check; G x E = Genotype by environment interaction; LSD = Least significant difference, CV = coefficient of variation

**Supplementary Table 3.** Grain yield of fall armyworm tolerant and local check hybrids under insecticide sprayed conditions across eight locations for on-station experiments

| Entry | GY | YAD | DA | DS | ASI | PH | EH | SL | HC | PA | EA | Rot | EPP |
| --- | --- | --- | --- | --- | --- | --- | --- | --- | --- | --- | --- | --- | --- |
| FAWTH1 | 7225.29 | 18.47 | 53.2 | 54.0 | 1.1 | 216.78 | 110.22 | 17.43 | 1.86 | 2.67 | 2.61 | 2.56 | 0.96 |
| FAWTH2 | 7549.49 | 23.79 | 53.6 | 54.1 | 0.9 | 219.00 | 111.44 | 18.30 | 1.61 | 2.78 | 2.67 | 2.22 | 0.95 |
| FAWTH3 | 8441.24 | 38.41 | 53.2 | 54.1 | 1.0 | 212.39 | 106.11 | 11.94 | 1.83 | 2.58 | 2.28 | 2.17 | 0.95 |
| Opeaburo | 6098.86 | 0.00 | 53.4 | 54.3 | 1.0 | 203.78 | 103.44 | 10.39 | 1.94 | 2.72 | 3.22 | 2.56 | 0.93 |
| R-Square | 0.97 |  | 0.5 | 0.4 | 0.6 | 0.79 | 0.69 | 0.60 | 0.48 | 0.55 | 0.82 | 0.80 | 0.44 |
| CV (%) | 6.22 |  | 3.1 | 2.5 | 48.9 | 5.45 | 8.25 | 95.08 | 19.05 | 21.59 | 17.68 | 44.66 | 12.91 |
| LSD | 455.51 |  | 1.7 | 1.4 | 0.5 | 11.60 | 8.89 | 13.80 | 0.35 | 0.58 | 0.48 | 1.06 | 0.12 |
| Mean | 7328.72 |  | 53.4 | 54.1 | 1.0 | 212.99 | 107.81 | 14.51 | 1.81 | 2.69 | 2.69 | 2.38 | 0.95 |
| Location | *** |  | ns | ns | ns | *** | * | ns | ns | ns | *** | ** | ns |
| Entry | *** |  | ns | ns | ns | ** | * | ns | ns | ns | *** | ns | ns |
| G x E | ** |  | ns | ns | ns | ** | * | ** | ns | ns | *** | ns | ns |
| Heritability | 0.97 |  |  |  |  | 0.75 |  |  | 0.77 |  | 0.76 |  |  |

**†** GY= Grain yield (kg ha^-1^); DA = days to 50% pollen shed; DS = Days to 50% silking; ASI-anthesis silking interval; PH = plant height; EH-ear height; PA = plant aspect; EA-ear aspect; EPP-ears per plant; HC = husk cover; Rot = ear rot; EA-ear aspect; RL = root lodging, SL = Stalk lodging; *P≤0.05, **P≤0.01, P≤0.001, ns = not significant; YAD = yield advantage over the commercial check; G x E = Genotype by environment interaction; LSD = Least significant difference, CV = coefficient of variation

**Supplementary Table 4.** On-farm grain yield (kg ha^-1^) of three fall armyworm tolerant hybrids and a local check evaluated at 10 locations in Ghana

| Genotype | Fumesua | Damongo | Ho | Dzakiti | Manga | Naajon | Nyankpala | Tumu | Wa | Yendi |
| --- | --- | --- | --- | --- | --- | --- | --- | --- | --- | --- |
| FAWTH1 | 5954.69 | 6784.50 | 6869.27 | 6921.86 | 3907.30 | 6386.89 | 6491.23 | 7193.81 | 6700.13 | 7369.36 |
| FAWTH2 | 6075.69 | 6546.70 | 6733.36 | 5867.45 | 3768.10 | 6097.30 | 5635.21 | 6264.87 | 6461.30 | 6320.78 |
| FAWTH3 | 7931.01 | 8830.35 | 7404.87 | 8547.38 | 4580.12 | 7116.21 | 7534.50 | 8325.96 | 7854.50 | 8614.55 |
| Opeaburo | 4572.51 | 5015.27 | 3338.17 | 2927.09 | 1007.23 | 2079.97 | 1729.63 | 5190.77 | 3754.50 | 4420.24 |
| Heritability | 0.94 | 0.98 | 0.99 | 0.98 | 0.97 | 0.98 | 0.99 | 0.97 | 0.98 | 0.99 |
| G. Variance | 2027432.99 | 2494496.07 | 3470233.93 | 5684903.86 | 2501396.07 | 5231435.16 | 6474636.57 | 1836167.58 | 3018221.37 | 3196459.00 |
| R. Variance | 404473.82 | 113417.67 | 88883.45 | 277203.89 | 21791.46 | 272469.10 | 162436.61 | 166356.98 | 18695.05 | 138110.87 |
| Mean | 6133.47 | 6794.25 | 6086.42 | 6065.95 | 3315.69 | 5420.09 | 5347.64 | 6743.85 | 6192.60 | 6681.23 |
| LSD | 1230.37 | 667.80 | 593.11 | 1043.45 | 294.50 | 1033.94 | 801.87 | 802.85 | 272.89 | 737.19 |
| CV | 10.37 | 4.96 | 4.90 | 8.68 | 4.45 | 9.63 | 7.54 | 6.05 | 2.21 | 5.56 |

**†** LSD = Least significant difference, CV = coefficient of variation; G variance = genotypic variance; R variance = residual variance
